# Flowers in Conservation Reserve Program (CRP) Pollinator Plantings and the Upper Midwest Agricultural Landscape Supporting Honey Bees

**DOI:** 10.3390/insects11070405

**Published:** 2020-06-30

**Authors:** Harper McMinn-Sauder, Rodney Richardson, Tyler Eaton, Mike Smith, Reed Johnson

**Affiliations:** 1 Department of Entomology, The Ohio State University, Columbus, OH 43210, USA; rtr87@yorku.ca (R.R.); eaton.160@osu.edu (T.E.); johnson.5005@osu.edu (R.J.); 2 Conservation Technology Information Center, West Lafayette, IN 47906, USA; smith@ctic.org

**Keywords:** pollen, *Apis mellifera*, *Glycine*, Trifolium, Symphiotrichum, citizen science, CP42, DNA metabarcoding

## Abstract

A present goal of the Conservation Reserve Program (CRP) is to manage land in agricultural landscapes to increase pollinator abundance and diversity. CP42, or the pollinator seed mix, is planted and managed to support foraging pollinators with blooming flowers present at all points in the foraging season. This high-quality habitat provides an excellent opportunity to study honey bee nutrition and determine whether honey bees located near CRP sites use known resources included in planting seed mixes. This study aims to highlight the primary sources of honey bee forage in the northern Midwest as well as to assess honey bee utilization of the floral resources provided by the pollinator seed mix used for CRP plantings. We received pollen samples collected using pollen traps by beekeepers in Ohio, South Dakota, Indiana, Illinois, and Michigan. Metabarcoding methods were used to identify and quantify pollen collected at different points in the season. The results indicate that honey bees frequently used major mass flowering resources such as *Glycine, Trifolium,* and *Symphiotrichum* throughout the season. In addition, flowers included in the CRP pollinator seed mix were used modestly. These results have implications for pollinator seed mix design.

## 1. Introduction

The Conservation Reserve Program (CRP) was introduced in 1985 under the Food Security Act to promote the conversion of cropland with highly erodible soils into long-term conservation habitat [1,2,3,4]. While accomplishing this goal, the CRP has provided a number of positive ecological impacts, including increased landscape diversity and reduced habitat fragmentation [2,5]. A recent priority of the CRP has been to manage land specifically for the promotion of forage for pollinators [6]. To achieve this, the CP42 seed mix was designed for pollinator enrichment plantings. This mix is characterized by a high diversity of native forbs and grasses. By incorporating an array of flowers that bloom throughout the season, this management strategy aims to ensure that foraging pollinators have season-long access to pollen and nectar resources [7].

Managed honey bees, like all pollinators, have nutritional requirements that must be met by their surroundings in the form of pollen and nectar from flowers. As the sole source of protein for the colony, pollen is essential for normal physiological development and brood production [8,9,10]. Without sufficient pollen stores, the colony will decline in population [11,12,13]. In contrast to nectar, which is stored in the form of honey, pollen stores are limited, and pollen foraging effort is increased to meet colony demand [14,15,16,17]. This makes it essential that honey bees are located in a landscape that provides consistent floral resources with high quality pollen [18]. In addition to the need for the steady availability of pollen resources, a number of studies have demonstrated benefits to the colony from a diverse pollen diet [11,19,20,21]. Low diversity pollen increases the likelihood of colony loss due to declining immune system function resulting from dietary stress [13,19,22,23]. Having access to an assortment of summer pollen and nectar sources on diverse CRP summering grounds helps colonies build strength for survival through winter months [24,25].

Due to the improved forage that CRP-enrolled land provides, it is frequently sought out by migratory commercial beekeepers for the placement of honey bee colonies to recuperate from the stresses of crop pollination [26,27]. Commercially managed colonies travel to pollinate crops such as almonds and blueberries and often spend much of the foraging season under contract for pollinating monocultures [28]. This makes spending time outside of contracted pollination in locations with diverse and abundant floral resources important for sustaining healthy colonies. The upper Midwest is one region that is heavily cropped and holds large areas of CRP [29].

Though research has demonstrated the high value of forage provided by CRP to honey bee colonies, there is little direct evidence identifying the floral resources that are most valuable to bees in these landscapes [24,25,30,31]. While there are a number of ways to quantify honey bee pollen foraging habits, the use of metabarcoding to identify the floral origin of pollen has emerged as an increasingly effective method [32,33]. This method has enabled researchers to connect pollen composition with spatial foraging patterns and landscape structure [33,34]. This study aims to identify the main sources of pollen collected by honey bee colonies located near CRP plantings in the northern Midwest. In addition, we assess the extent of honey bee utilization of flowers included in the pollinator seed mix. 

## 2. Materials and Methods

### 2.1. Sample Collection

Pollen was collected from colonies managed by volunteer beekeepers with apiaries located in Ohio, Indiana, Illinois, South Dakota, and Michigan near CRP-enrolled land planted with pollinator seed mixes. Pollinator seed mixes sown at each site were region-specific and constituted a subset of the CP42 seed mix. A list of genera included in all site plantings is listed in Table 1. Samples were collected using bottom-mounted Sundance II pollen traps (Ross Rounds, Albany, NY, USA) approximately once per month over the duration of the season (May–October) in 2016 and 2017. Seventeen pollen samples in 2016, between 13 May and 25 October, and 13 pollen samples in 2017, between 1 May and 31 October, were collected. In 2016, four samples were collected from Ohio, Indiana, and Illinois, and five samples were collected from South Dakota. In 2017, three samples were collected from Ohio, eight samples were collected from Indiana, and two samples were collected from Michigan. Samples from beekeepers were sent via postal mail and stored at −20 °C upon arrival.

### 2.2. Pollen Resource Identification

The metabarcoding methods followed those of Richardson et al. [33]. For subsampling, pollen was homogenized by adding the lesser amount of either 20 g or 10% by mass of the sample to distilled water to yield a final concentration of 0.1 g/mL of pollen. This mixture was placed in a blender (Hamilton Beach #54225, Southern Pines, NC, USA) and homogenized for 2.5 minutes. Pollen homogenate, 1.4 mL containing approximately 140 mg of pollen, was then placed in a 2mL microcentrifuge tube (Fisherbrand Free-Standing Microcentrifuge Tubes; Fisher Scientific, Hampton, NH, USA) and zirconia beads (0.7mm; Fisher Scientific, Hampton, NH, USA) were added until a total volume of 1.5 mL was reached. Pollen was mechanically disrupted using a BeadBeater (Mini-BeadBeater-1; BioSpec Products, Bartlesville, OK, USA). A three-step PCR method was used to amplify regions in the nuclear *rbcL* and *trnL* genes and the ribosomal ITS2 region [35,36,37,38,39]. Using this method, 1 uL of sample DNA was used as the template for the first PCR step, while the template for the next two steps was 1 uL of PCR reaction from the previous step. The primers used in the first PCR step were universal primers for the respective barcode sequences. The primers in the second step appended template priming oligonucleotides to the amplicons from the first step, while primers in the third step were modified with sample indexing and lane hybridization oligonucleotides which were appended to the amplicons in the second step. Primer sequences and PCR conditions for each step are presented in Supplemental Table S1. Library preparation and purification steps were performed following the methods of Richardson et al. [33]. Pooled samples were sequenced at the Molecular and Cellular Imaging Center (MCIC) in Wooster, Ohio using an Illumina MiSeq cell (2 × 300 cycles) with the addition of 20% *phi*X.

Reference sequence libraries for *rbcL, trnL,* and ITS2 were downloaded from NCBI (accessed on 26 February 2019) and filtered to only include sequences from plants present in the five states included in this study (United States Department of Agriculture Plants Database (https://plants.usda.gov)). Reference sequences were processed using MetaCurator [40], a software package which relies on MAFFT [41], VSEARCH [42], and HMMER [43] to identify and extract the precise amplicon region of interest and dereplicate the resulting sequences using taxonomically aware methods. Sample sequence text files were imported to the Ohio Supercomputer Center remote computing system [44]. Forward and reverse reads were merged for *rbcL* and ITS2 regions. Due to marker sequence lengths, *trnL* sequences were left unmerged. ITS2 and *rbcL* sequences were then compared to reference sequences using semi-global VSEARCH alignment, requiring 98% query coverage, 92.5% sequence identity for ITS2, and 96% sequence identity for *rbcL*. Forward *trnL* reads were converted from fastq to fasta format and compared to reference *trnL* sequences using semi-global VSEARCH alignment. Top-scoring VSEARCH alignment matches were then annotated using the taxonomic lineages provided through the NCBI Taxonomy database and obtained using the taxonomizr R package [45]. Following taxonomic annotation, the data were then analyzed according to a consensus-filtered, median-based analysis [33]. Genus-level richness was calculated for each collected sample. In addition, a genus-level evenness analysis was conducted using average proportional abundances of taxa by year. Evenness was calculated by dividing the Shannon–Weiner diversity index value (H) by the natural log of the sample count of a given year.

## 3. Results

Following alignment, we considered all taxa that were detected in proportional abundances of 0.001% or greater. Proportional abundance represents the amount of a given taxa present within a single sample, relative to the other taxa detected in that sample. The consensus-filtered, median-based analysis yielded 104 genera. Many of these taxa were detected in very small quantities (<1%), therefore, a cutoff of 5% proportional abundance was established for further analysis.

### 3.1. Pollen Identification

**Figure 1:**
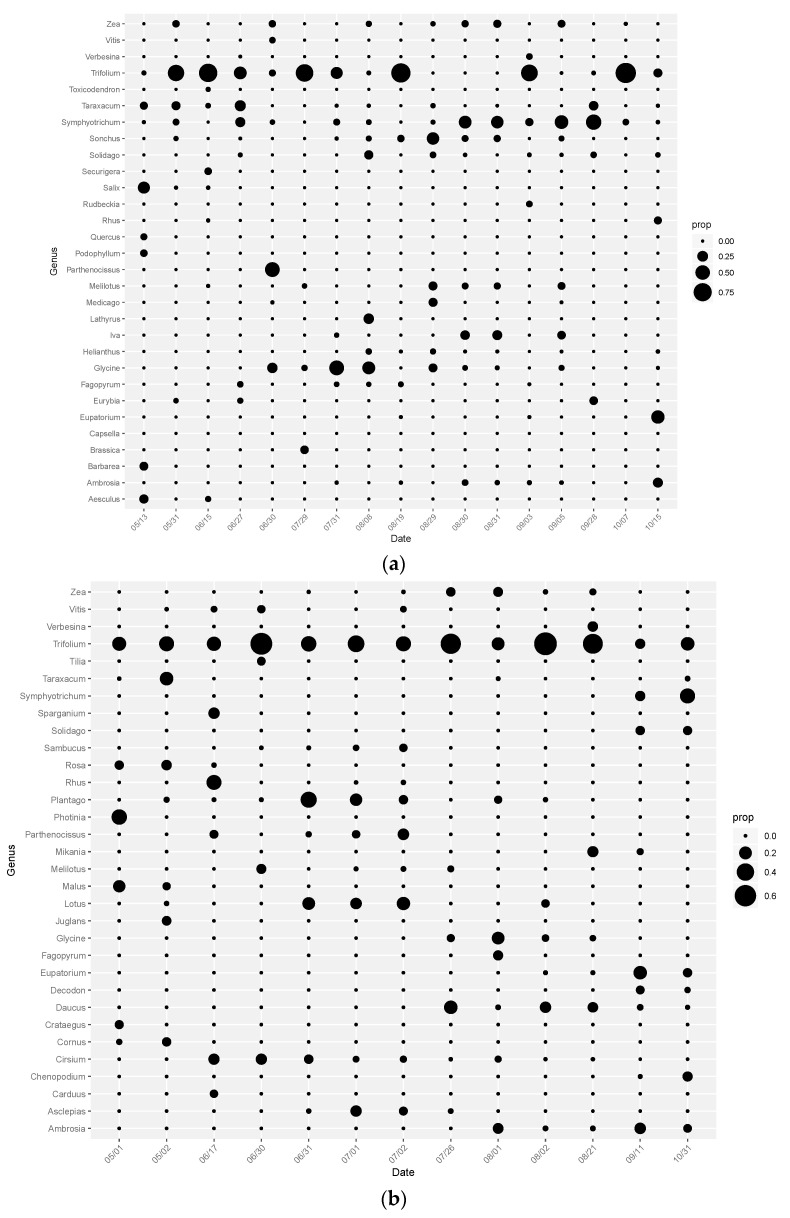
Proportional abundance of pollen samples using pollen metabarcoding analysis. Circle size indicates proportional abundance of a given taxa on a given sampling date. (**a**) In 2016, key genera detected were *Trifolium* (clovers), *Symphyotrichum* (American asters), and *Glycine* (soybeans). (**b**) In 2017, *Trifolium* was a key genus detected; however, a diverse array of additional taxa were detected in moderate abundances.

Honey bees located near CRP in the northern Midwest were found to collect pollen from a large number of genera in abundances >5%. In 2016, pollen from 30 genera and 11 families were detected at >5% abundance (Figure 1). Genus-level richness of pollen samples ranged from 3 to 11, with a mean value of 7.56 (STDEV = 2.1) in 2016. An analysis of genera evenness within 2016 samples indicates an evenness value of 0.72 (Supplemental Table S2). In 2017, pollen from 32 genera and 17 families were detected at >5% abundance. Genus-level richness ranged from 7 to 11, with a mean value of 8.62 (STDEV = 1.12) in 2017. The analysis of evenness of the 2017 samples indicates an evenness value of 0.72 (Supplemental Table S2). In 2016, the most highly utilized genera were *Trifolium* (clovers), *Symphyotrichum* (American asters), and *Glycine* (soybean). *Trifolium* was detected in moderate to high (>50%) proportional abundances throughout most of the season in 2016 and throughout the entire season in 2017. In addition to *Trifolium*, other weedy forbs also were collected in noteworthy quantities. *Taraxacum* (dandelion) was detected in abundances >5% early in the season in both 2016 and 2017. In 2016, *Symphyotrichum* was detected at 25–50% abundance from the end of August through October. *Glycine* was detected in high abundance primarily in July. In addition to forbs, flowering trees and shrubs were detected in high abundance throughout the season, but particularly in Spring. *Salix* (willow) and *Quercus* (oak) were detected in high abundance in 2016 and *Malus* (apple) and *Rhus* (sumac) were found early in the season in 2017. Raw proportional abundance values are reported in Supplemental Tables S3 and S4.

### 3.2. Pollinator Seed Mix Utilization

Of the 15 genera included in the CP42 pollinator seed mix, four genera were detected in pollen from the 2016 and 2017 samples (Table 1). Three of the four detected genera are in the Asteraceae (*Aster, Heliopsis and Rudbeckia*) and the fourth genus belongs to the family Fabaceae (*Trifolium*). In 2016, two genera *Trifolium* (clovers) and *Rudbeckia* (coneflowers) were detected in abundances >5%. One genus, *Aster*, was detected in abundances <5% in nine samples. *Trifolium* was the only flower in the CP42 seed mix that was detected in abundances >5% throughout the sampling period. *Aster*, *Heliopsis* (false sunflower), and *Rudbeckia* were detected in abundances <5% in five samples throughout the season in 2017.

## 4. Discussion

In both 2016 and 2017, mixed pollen samples collected from honey bee colonies located near CRP pollinator plantings contained moderate abundances of a diverse array of pollen from both these plantings and elsewhere in the agricultural landscape. These results demonstrate the importance of opportunistic forbs, such as *Trifolium* (clover), and *Taraxacum* (dandelion) for honey bee pollen foraging throughout the season. Like these common weeds, native plants with similar life-histories, such as *Solidago* (goldenrod), have the ability to colonize and grow at high densities in unmanaged landscapes [46,47,48]. While many seek to eradicate these native and non-native forbs from highly managed landscapes, such as cropland and CRP, these plants play an important role in the diet of honey bees [5,49,50]. This is likely due to the great abundance these species can achieve in agricultural landscapes where they are commonly found on roadsides, crop edges, and interstitial areas [51,52,53]. In addition to opportunistic forbs, mass flowering trees and shrubs can provide resources during early spring [5,32,33,54,55]. In this study, we found high proportional abundance of *Salix* (willow) and *Quercus* (oak) in 2016, and *Malus* (apple) and *Rhus* (sumac) in 2017. Flowering trees and shrubs are often heavily foraged upon due to the large quantity of floral resources, especially pollen, that they produce [56]. One commonly detected pollen in 2016 was *Glycine* (soybean), supporting an existing body of research highlighting the potential value of cultivated soybean in the honey bee diet [57,58,59,60]. Areas enrolled in CRP in the northern Midwest are generally embedded within agricultural landscapes consisting of corn and soybean fields. While soybeans may provide a mid-season mass flowering resource during bloom, there is also potential for pesticide exposure during planting and through the season [61,62]. One resource that was measured in lower abundances than expected in both 2016 and 2017 was *Solidago* (goldenrod), a typically important late-season foraging resource for honey bees in the Midwest [63,64,65]. This result may be due to technical limitations in distinguishing among different genera within the Asteraceae using *rbcL* and *trnL*. To address this issue, future studies involving *Solidago* should use markers with greater variability that should be better able to distinguish genera within the Asteraceae, such as *matK* or ITS1 [66,67,68].

Four of the 15 plant genera present in the CP42 pollinator seed mix contributed to honey bee pollen collection in this study. The majority of these genera were detected in very small quantities. This either indicates that many of the species present in the mix are not used by honey bees for pollen collection or that their abundance in the landscape is low. *Trifolium* (clover) was commonly detected at moderate to high levels, demonstrating that this is a key pollen floral resource for honey bees. The sampling of multiple states in this study limits our ability to draw conclusions regarding the universal suitability of CP42, as there is regional variation in seed mixes. The incorporation of pollinator-friendly forbs such as *Dalea* (prairie clovers) or *Monarda* (beebalms) could make this mix better suited for foraging honey bees as well as native pollinators in the northern Midwest [69]. The addition of multiple species in highly attractive genera such as *Trifolium* and *Solidago* could also increase honey bee utilization through the provision of late season foraging resources [52]. It is likely that some genera included in the pollinator seed mix are maximally attractive to specialist pollinators and were included in the seed mix to support pollinator diversity. The lack of detection of other flowers included in the seed mix could also be driven by lack of germination of particular seeds, as we did not perform floral assessments to identify plants present within study sites. In addition, the size and abundance of CRP sites within the foraging range of each apiary is unknown, suggesting that some apiaries may have had high abundance of CRP within their landscape while others may have had very little. These variables along with unbalanced spatial and temporal sampling could be potential drivers of trends in the analysis of pollen resource use. Future research should emphasize frequent sampling and uniformity in planting mix and local CRP landcover proportion. Though these seed mixes are designed with pollinators in mind, it is unrealistic to expect a seed mix to accurately mimic the ideal foraging landscape [70]. Further research will be necessary to better understand the key floral resources needed to support honey bees and how these plants can be incorporated into the design of future seed mixes.

## 5. Conclusions

This study identifies the floral resources most heavily utilized for pollen collection by honey bee colonies located near CRP pollinator plantings in the northern Midwest. The high diversity of plant taxa detected in the metabarcoding analysis indicates that bees located near CRP have a variety of flowers available for pollen collection from across the agricultural landscape. As land enrolled in CRP is deeply embedded within the agroecosystem, this presents an opportunity to potentially enrich this region to support foraging pollinators. Mass flowering plants, such as *Trifolium, Symphyotrichum,* and *Glycine*, play a key role for honey bees located in agricultural regions. In addition, the modest pollen collection observed from species included in the pollinator seed mix suggests that there is room for improvement in the design of these seed mixes to maximize honey bee usage, though additional studies utilizing non-CRP control plots are needed to validate this.

## Figures and Tables

**Table 1:** Plant families and genera included in the CP42 pollinator seed mix. Bold type indicates plants detected in pollen samples collected by honey bees.

Family	Genus	Family	Genus	Family	Genus
**Asteraceae** Asteraceae Asteraceae Asteraceae **Asteraceae**	***Aster*** *Coreopsis* *Dalea* *Echinaceae* **Heliopsis**	Asteraceae **Asteraceae** Fabaceae Fabaceae Fabaceae	*Ratibida* ***Rudbeckia*** *Amorpha* *Cassia* *Chamaecrista*	Fabaceae Fabaceae **Fabaceae** Poaceae Poaceae	*Desmanthus* *Desmodium* ***Trifolium*** *Andropogon* *Bouteloua*

## Supplementary Materials

The following are available online at https://www.mdpi.com/2075-4450/11/7/405/s1, Table S1: Primer sequences for PCR amplification of pollen samples, Table S2: Diversity statistics for pollen metabarcoding samples, Table S3: Proportional abundance of taxa detected in pollen samples in 2016, Table S4; Proportional abundance of taxa detected in pollen samples in 2017.
