# Supplementary material for: Flowers in Conservation Reserve Program (CRP) Pollinator Plantings and the Upper Midwest Agricultural Landscape Supporting Honey Bees
Source: Insects. 2020 Jun 30;11(7):405. doi: 10.3390/insects11070405 (PMC7411617; doi:10.3390/insects11070405)
Supplement: Supplementary file 1 [file insects-11-00405-s001.pdf]

Supplemental Materials

Table 1. Primer sequences for PCR amplification of pollen samples.

Marker	Primer Name	Primer Sequence	Primer Concentration	Cycles	Time	Additives	Reaction Volume
RBCL 1	SH_rbcl2	TGGCAGCATTYCGAGTAACTC	0.5 uM	20	58	0.8uL BSA, 1 uL DMSO	20 uL
RBCL 1	SH_rbcla-R	GTAAAATCAAGTCCACCRCG	0.5 uM	20	58	0.8 uL BSA, 1uL DMSO	20 uL
TRNL 1	trnLUAA_F	CGAAATCGGTAGACGCTACG	0.5 uM	25	58	0.8 uL BSA, 1 uL DMSO	20 uL
TRNL 1	trnLUAA_R	GGGGATAGAGGGACTTGAAC	0.5 uM	25	58	0.8 uL BSA, 1 uL DMSO	20 uL
ITS2 1	ITS-S2F	ATGCGATACTTGGTGRGAAT	0.5 uM	20	60	0.8 uL BSA, 1 uL DMSO	20 uL
ITS2 1	ITS4R	TCCTCCGCTTATTGATATGC	0.5 uM	20	60	0.8 uL BSA, 1 uL DMSO	20 uL
RBCL 2	SH_rbcL2_P2	TCGTCGGCAGCGTCAGATGTGTATAAGAGACAGTGGCAGCATTYCGAGTAACTC	0.1 uM	15	50.2	0.8 uL BSA, 1 uL DMSO	20 uL
RBCL 2	SH_rbcLa-R_P2	GTCTCGTGGGCTCGGAGATGTGTATAAGAGACAGGTAAAATCAAGTCCACCRCG	0.1 uM	15	50.2	0.8 uL BSA, 1 uL DMSO	20 uL
TRNL 2	trnLUAA_P2_F	TCGTCGGCAGCGTCAGATGTGTATAAGAGACAGCGAAATCGGTAGACGTACG	0.1 uM	18	55.4	0.8 uL BSA, 1 uL DMSO	20 uL
TRNL 2	trnLUAA_P2_R	GTCTCGTGGGCTCGGAGATGTGTATAAGAGACAGGGGGATAGAGGGACTTGAAC	0..1 uM	18	55.4	0.8 uL BSA, 1 uL DMSO	20 uL
ITS2 2	PCR2_ITS2_f	TCGTCGGCAGCGTCAGATGTGTATAAGAGACAGATGCGATACTTGGTGTGAAT	0.1 uM	15	52.3	0.8 uL BSA, 1 uL DMSO	20 uL
ITS2 2	PCR2_ITS2_r	GTCTCGTGGGCTCGGAGATGTGTATAAGAGACAGTCCTCCGCTTATTGATATGC	0.1 uM	15	52.3	0.8 uL BSA, 1 uL DMSO	20 uL
	PCR3_S502_f	AATGATACGGCGACCACCGAGATCTACACCTCTCTATTCGTCGGCAGCGTC	0.5 uM	25	66		20 uL
	PCR3_S503_f	AATGATACGGCGACCACCGAGATCACACTATCCTCTTCGTCGGCAGCGTC	0.5 uM	25	66		20 uL
	PCR3_S505_f	AATGATACGGCGACCACCGAGATCTACACGTAAGGAGTCGTCGGCAGCGTC	0.5 uM	25	66		20 uL
	PCR3_S506_f	AATGATACGGCGACCACCGAGATCTACACACTGCATATCGTCGGCAGCGTC	0.5 uM	25	66		20 uL
	PCR3_S507_f	AATGATACGGCGACCACCGAGATCTACACAAGGAGTATCGTCGGCAGCGTC	0.5 uM	25	66		20 uL
	PCR3_S508_f	AATGATACGGCGACCACCGAGATCTACACCTAAGCCTTCGTCGGCAGCGTC	0.5 uM	25	66		20 uL
	PCR3_S510_f	AATGATACGGCGACCACCGAGATCTACACCGTCTAATTCGTCGGCAGCGTC	0.5 uM	25	66		**20 uL**
	PCR3_S511_f	AATGATACGGCGACCACCGAGATCTACACTCTCTCCGTCGTCGGCAGCGTC	0.5 uM	25	66		**20 uL**

PCR3_s513_f	AATGATACGGCGACCACCGAGATCTACACTCGACTAGTCGTCGGCAGCGTC	0.5 uM	25	66	**20 uL**
PCR3_s515_f	AATGATACGGCGACCACCGAGATCTACACTTCTAGCTTCGTCGGCAGCGTC	0.5 uM	25	66	20 uL
PCR3_s516_f	AATGATACGGCGACCACCGAGATCTACACCCTAGAGTTCGTCGGCAGCGTC	0.5 uM	25	66	20 uL
PCR3_s517_f	AATGATACGGCGACCACCGAGATCTACACGCGTAAGATCGTCGGCAGCGTC	0.5 uM	25	66	20 uL
PCR3_s518_f	AATGATACGGCGACCACCGAGATCTACACCTATTAAGTCGTCGGCAGCGTC	0.5 uM	25	66	20 uL
PCR3_s520_f	AATGATACGGCGACCACCGAGATCTACACAAGGCTATTCGTCGGCAGCGTC	0.5 uM	25	66	20 uL
PCR3_N701_r	CAAGCAGAAGACGGCATACGAGATTCGCCTTAGTCTCGTGGGCTCGG	0.5 uM	25	66	20 uL
PCR3_N702_r	CAAGCAGAAGACGGCATACGAGATCTAGTACGGTCTCGTGGGCTCGG	0.5 uM	25	66	20 uL
PCR3_N703_r	CAAGCAGAAGACGGCATACGAGATTTCTGCCTGTCTCGTGGGCTCGG	0.5 uM	25	66	20 uL
PCR3_N704_r	CAAGCAGAAGACGGCATACGAGATGCTCAGGAGTCTCGTGGGCTCGG	0.5 uM	25	66	20 uL
PCR3_N705_r	CAAGCAGAAGACGGCATACGAGATAGGAGTCCGTCTCGTGGGCTCGG	0.5 uM	25	66	20 uL
PCR3_N706_r	CAAGCAGAAGACGGCATACGAGATCATGCCTAGTCTCGTGGGCTCGG	0.5 uM	25	66	20 uL
PCR3_N707_r	CAAGCAGAAGACGGCATACGAGATGTAGAGAGGTCTCGTGGGCTCGG	0.5 uM	25	66	20 uL
PCR3_N710_r	CAAGCAGAAGACGGCATACGAGATCAGCCTCGGTCTCGTGGGCTCGG	0.5 uM	25	66	20 uL
PCR3_N711_r	CAAGCAGAAGACGGCATACGAGATTGCCTCTTGTCTCGTGGGCTCGG	0.5 uM	25	66	20 uL
PCR3_N712_r	CAAGCAGAAGACGGCATACGAGATTCCTCTACGTCTCGTGGGCTCGG	0.5 uM	25	66	20 uL
PCR3_N714_r	CAAGCAGAAGACGGCATACGAGATTCATGAGCGTCTCGTGGGCTCGG	0.5 uM	25	66	20 uL

	PCR3_N715_r	CAAGCAGAAGACGGCATACGAGATCCTGAGATGTCTCGTGGGCTCGG	0.5 uM	25	66	20 uL
	PCR3_N716_r	CAAGCAGAAGACGGCATACGAGATTAGCGAGTGTCTCGTGGGCTCGG	0.5 uM	25	66	20 uL
	PCR3_N718_r	CAAGCAGAAGACGGCATACGAGATGTAGCTCCGTCTCGTGGGCTCGG	0.5 uM	25	66	20 uL

Table 2. Diversity statistics for pollen metabarcoding samples.

Year	Genera Detected	Species Richness	Evenness
2016	28	7.56 (STDEV = 2.1)	0.72
2017	32	8.62 (STDEV = 1.12)	0.72

Table 3. Proportional abundance of taxa detected in pollen samples in 2016.

Genus	5/13	5/31	6/15	6/27	6/30	7/29	7/31	8/8	8/19	8/29	8/30	8/31	9/3	9/5	9/28	10/7	10/15
Aesculus	0.17	0	0.05	0	0	0	0	0	0	0	0	0	0	0	0	0	0
Ambrosia	0	0	0	0	0	0	0.01	0	0.01	0	0.06	0.02	0.02	0.01	0	0	0.21
Barbarea	0.15	0	0	0	0	0	0	0	0	0	0	0	0	0	0	0	0
Brassica	0	0	0	0	0	0.14	0	0	0	0	0	0	0	0	0	0	0
Eupatorium	0	0	0	0	0	0	0	0	0	0	0	0	0	0	0	0	0.42
Eurybia	0	0.03	0	0.04	0	0	0	0	0	0	0	0	0	0	0.14	0	0
Fagopyrum	0	0	0	0.06	0	0	0.03	0.02	0.04	0	0	0	0	0	0	0	0
Glycine	0	0	0	0	0.24	0.05	0.52	0.38	0	0.15	0.03	0.02	0	0.04	0	0	0
Helianthus	0	0	0	0	0	0	0	0.05	0	0.04	0	0	0	0	0	0	0.01
Iva	0	0	0	0	0	0	0.02	0	0	0	0.18	0.21	0	0.14	0	0	0
Lathyrus	0	0	0	0	0	0	0	0.24	0	0	0	0	0	0	0	0	0
Medicago	0	0	0	0	0.01	0	0	0	0	0.16	0	0	0	0	0	0	0
Melilotus	0	0	0.01	0	0	0.03	0	0	0	0.15	0.07	0.07	0	0.10	0	0	0
Parthenocissus	0	0	0	0	0.52	0	0	0	0	0	0	0	0	0	0	0	0
Podophyllum	0.09	0	0	0	0	0	0	0	0	0	0	0	0	0	0	0	0
Quercus	0.07	0	0	0	0	0	0	0	0	0	0	0	0	0	0	0	0
Rhus	0	0	0.01	0	0	0	0	0	0	0	0	0	0	0	0	0	0.11
Rudbeckia	0	0	0	0	0	0	0	0	0	0	0	0	0.06	0	0	0	0
Salix	0.33	0.01	0.01	0	0	0	0	0	0	0	0	0	0	0	0	0	0
Securigera	0	0	0.10	0	0	0	0	0	0	0	0	0	0	0	0	0	0
Solidago	0	0	0	0.02	0	0	0	0.17	0	0.06	0.01	0	0.01	0.01	0.06	0	0.02
Sonchus	0	0.02	0	0	0	0	0.01	0.04	0.09	0.36	0.07	0.08	0	0.04	0	0	0
Symphyotrichum	0.01	0.07	0	0.21	0.03	0	0.07	0.03	0	0.02	0.38	0.36	0.12	0.44	0.45	0.06	0.01
Taraxacum	0.11	0.16	0.08	0.27	0	0	0	0.01	0	0.03	0	0	0	0	0.19	0	0.01
Trifolium	0.02	0.63	0.77	0.38	0.07	0.72	0.33	0.01	0.84	0	0	0	0.66	0	0.01	0.91	0.16
Verbesina	0	0	0	0	0	0	0	0	0	0	0	0	0.06	0	0	0	0
Vitis	0	0	0	0	0.06	0	0	0	0	0	0	0	0	0	0	0	0
Zea	0	0.09	0	0	0.09	0	0	0.05	0	0.02	0.08	0.12	0	0.10	0	0.01	0

Table 4. Proportional abundance of taxa detected in pollen samples in 2017.

Genus	5/1	5/2	6/17	6/30	6/31	7/1	7/2	7/26	8/1	8/2	8/21	9/11	10/31
Ambrosia	0	0	0	0	0	0			0.13	0.01	0.01	0.15	0.06
Asclepias	0	0	0	0	0.01	0.15	0.07	0.01	0	0	0	0	0
Carduus	0	0	0.06	0	0	0	0	0	0	0	0	0	0
Chenopodium	0	0	0	0	0	0	0	0	0	0	0	0.01	0.10
Cirsium	0	0	0.14	0.14	0.08	0.03	0.03	0	0.03	0	0	0	0
Cornus	0.02	0.08	0	0	0	0	0	0	0	0	0	0	0
Crataegus	0.08	0	0	0	0	0	0	0	0	0	0	0	0
Daucus	0	0	0	0	0	0	0	0.23	0.01	0.14	0.12	0.02	0.01
Decodon	0	0	0	0	0	0	0	0	0	0	0	0.07	0.02
Eupatorium	0	0	0	0	0	0	0	0	0	0.01	0.01	0.23	0.08
Fagopyrum	0	0	0	0	0	0	0	0	0.10	0	0	0	0
Glycine	0	0	0	0	0	0	0	0.05	0.19	0.04	0.02	0	0
Juglans	0	0.09	0	0	0	0	0	0	0	0	0	0	0
Lotus	0	0.01	0	0	0.19	0.15	0.22	0	0	0.06	0	0	0
Malus	0.18	0.06	0	0	0	0	0	0	0	0	0	0	0
Melilotus	0	0	0	0.10	0	0.01	0.01	0.03	0	0	0	0	0
Mikania	0	0	0	0	0	0	0	0	0	0	0.14	0.03	0
Parthenocissus	0	0	0.07	0	0.02	0.06	0.15	0	0	0	0	0	0
Photinia	0.31	0	0	0	0	0	0	0	0	0	0	0	0
Plantago	0	0.02	0	0	0.34	0.18	0.08	0	0.05	0	0	0	0
Rhus	0	0	0.28	0	0	0	0.01	0	0	0	0	0	0
Rosa	0.08	0.12	0.01	0	0	0	0	0	0	0	0	0	0
Sambucus	0	0	0	0	0.01	0.02	0.06	0	0	0	0	0	0
Solidago	0	0	0	0	0	0	0	0	0	0	0	0.08	0.08
Sparganium	0	0	0.15	0	0	0	0	0	0	0	0	0	0
Symphyotrichum	0	0	0	0	0	0	0	0	0	0	0	0.11	0.30
Taraxacum	0	0.23	0	0	0	0	0	0	0	0	0	0	0.01
Tilia	0	0	0	0.07	0	0	0	0	0	0	0	0	0
Trifolium	0.24	0.29	0.26	0.62	0.31	0.37	0.29	0.54	0.20	0.67	0.52	0.11	0.23
Verbesina	0	0	0	0	0	0	0	0	0	0	0.11	0	0
Vitis	0	0	0.03	0.06	0	0	0.02	0	0	0	0	0	0
Zea	0	0	0	0	0	0	0	0.09	0.10	0.01	0.03	0	0
